# Sex Differences in Efficacy and Safety After Left Atrial Appendage Closure: A 4.3-Year Follow-Up Analysis

**DOI:** 10.3389/fcvm.2022.814958

**Published:** 2022-05-18

**Authors:** Yuyi Chen, Yonghua Zhang, Lulu Qu, Chunyu Chen, Xi Su, Yanhong Chen

**Affiliations:** Department of Cardiology, Wuhan Asia Heart Hospital, Wuhan, China

**Keywords:** atrial fibrillation, left atrial appendage closure, gender, stroke, bleeding

## Abstract

**Introduction:**

Women are related to higher stroke risk and poorer outcome after stroke attack in patients with non-valvular atrial fibrillation (NVAF). The sex differences in efficacy and safety after left atrial appendage closure (LAAC) have remained elusive. This retrospective study aimed to investigate the safety, feasibility, and clinical outcomes of LAAC between women and men.

**Methods:**

From 2014 to 2018, 395 patients who underwent LAAC in our center were enrolled in this retrospective study. Baseline clinical characteristics, procedural parameters, and postoperative follow-up data were collected and compared between women and men.

**Results:**

The study included 154 women and 241 men. Compared with men, women were older (68.1 ± 7.9 vs. 64.6 ± 8.8, ***p*** < 0.01**), with higher CHA2DS2-VASc score (4.0 ± 1.7 vs. 3.0 ± 1.6, ***p*** < 0.01**). During the mean follow-up duration of 1,566 days (4.3 years), there were 39 major adverse cardiovascular events (MACE, including 19 cardiovascular or unexplained deaths, 15 ischemic strokes, and 5 major bleedings) recorded in 34 patients. The overall rate of ischemic stroke was 0.9 per 100 patient-years, and the overall rate of MACE was 2.1 per 100 patient-years. The cardiovascular or unexplained death and major bleeding were comparable between women and men. Compared with men, women had lower rates of ischemic stroke, fatal or disabling ischemic stroke, MACE, and fatal or disabling MACE, but none of them reached statistical differences (**HR:** 0.361, ***p*** = 0.099, **HR:** 0.429, ***p*** = 0.276, **HR:** 0.600, ***p*** = 0.170, and **HR:** 0.621, ***p*** = 0.254, respectively). In the adjusted analyses with multivariate Cox regression models, women had a lower fatal or disabling ischemic stroke rate compared with men (**HR:** 0.100, ***p*** = 0.041).

**Conclusion:**

Left atrial appendage closure was feasible and safe for patients of both genders. The ischemic stroke, cardiovascular or unexplained death, and major bleeding were comparable between women and men. However, women were the independent protective factors against fatal or disabling ischemic stroke after LAAC implantation.

## Introduction

Non-valvular atrial fibrillation (NVAF) is the most common arrhythmia, associated with the severe complication of ischemic stroke. There are differences between women and men in terms of presentation and management in all forms of NVAF-related stroke. Women with NVAF are generally older than men, with a higher stroke rate. They also have worse long-term outcomes after the stroke attack (1). Despite their higher stroke risk, women are less likely to be treated with anticoagulation therapy (2).

Autopsy and surgical data have demonstrated that 90% of atrial thrombus originated from the left atrial appendage (LAA) in patients with NVAF (3). LAA closure (LAAC) has emerged as an alternative for oral anticoagulants (OACs) among patients with NVAF with contraindication for long-term OACs or high bleeding risk. Despite the significant gender differences in prevalence and prognosis of NVAF-related stroke, there have been few comparisons of the clinical outcomes between female and male patients after LAAC operation. This retrospective study aimed to investigate the safety, feasibility, and clinical outcomes of LAAC between female and male patients in a real-world setting of Chinese mainland.

## Materials and Methods

### Study Population

This is a retrospective study and patients’ archived data were analyzed anonymously. The authors had no access to information that could identify individual participants during or after data collection. Consecutive patients who underwent LAAC from 2014 to 2018 were enrolled. The indications for LAAC implantation were as follows: patients were over 18 years old, presented with paroxysmal or persistent NVAF, with CHA2DS2-VASc score ≥ 1, plus one of the following situations: high bleeding risk (HAS-BLED score ≥ 3), contraindication or unwillingness to long-term warfarin/novel oral anticoagulants (NOACs), or suffering from stroke/TIA despite the regular anticoagulation therapy. Baseline clinical characteristics such as gender, age, hypertension, diabetes mellitus, coronary artery disease (CAD), congestive heart failure, ischemic stroke/TIA history, major bleeding history, CHA2DS2-VASc score, HAS-BLED score, abnormal renal function, abnormal liver function, and body mass index (BMI) were recorded for every patient. Left atrial diameter (LAD), left ventricular end diastolic diameter (LVEdD), and left ventricular ejection fraction (LVEF) were measured with transthoracic echocardiography (TTE). LAA orifice and length were measured with transesophageal echocardiography (TEE).

### Device Implantation Operations

Left atrial appendage closure devices including Watchman, Amplatzer Cardiac Plug (ACP), LAmbre, or Lefort were implanted through trans-septal approaches, using catheter-based delivery systems. Briefly, the operations were conducted under general anesthesia and tracheal intubation. After the TEE-guided atrial septum puncture, LAA angiography of the right anterior oblique (RAO) at 30^°^ plus caudal (CAU) at 20^°^ was performed for LAA measurements. Suitable plug devices chosen according to the LAA measurements were delivered through catheter-based delivery systems and expanded to close the LAA openings. During the procedures, TEE was performed to confirm the LAAC.

### In-Hospital Management and Follow-Up

After the operations, patients were transferred to the cardiac care unit (CCU) for anesthesia recovery. A TTE was performed at the day of the operation to rule out pericardial effusion or device-related embolism. Then, 2 or 3 days of observation were completed before the patients discharged from hospital.

After LAAC implantation, patients were treated with warfarin to maintain the INR ranging from 2.0 to 3.0. For some patients with warfarin contraindication or unwillingness to warfarin, NOAC or dual antiplatelet therapy (DAPT) was prescribed. At postoperative 45 days, TEE was performed to assess the residual flow, stability of the device, and device-related thrombosis (DRT). If TEE verified that LAA had been closed with residual flow of less than 5 mm and had no DRT, warfarin or NOAC was discontinued. Patients then took a combination of aspirin and clopidogrel for an additional 4.5 months. After that, patients were treated with long-term aspirin alone.

Patients would come to our center for the first outpatient follow-up at postoperative 45 days. Apart from the programmed clinical visit at postoperative 45 days, clinical visits or telephone interviews were carried out at postoperative 6 months and one time per year for each patient.

Major adverse cardiovascular events (MACEs) were defined as the composite of cardiovascular or unexplained death, embolic event (ischemic stroke or systemic embolism), or major bleeding event.

### Statistical Analysis

Data were presented with means and standard deviations (*SD*) for continuous variables or with n and percentages for categorical variables. Comparisons were made with independent sample *t*-tests for normal distributed data. Mann–Whitney U tests were used for comparisons of non-normal distributed data. Categorical data were compared using chi-square tests. Rates of cardiovascular or unexplained death/ischemic stroke/fatal or disabling ischemic stroke/major bleeding/MACE/fatal or disabling MACE were calculated as a number of events per 100 patient-years. Then, relative risk reductions (RRRs) of different clinical outcomes between female and male patients were calculated. For time-to-event data, Kaplan–Meier curves and log-rank tests were computed with GraphPad Prism software. Adjusted analyses for ischemic stroke, fatal or disabling ischemic stroke, MACE, and fatal or disabling MACE were performed with multivariate Cox regression analyses or time-dependent Cox regression analyses, considering potential confounding factors that combined *p* < 0.2 for comparisons between the two groups with clinical factors. *p*-values of less than 0.05 were considered significant. Statistical analyses were completed with SPSS v.25.0 statistical analysis software package.

## Results

**FIGURE 1:**
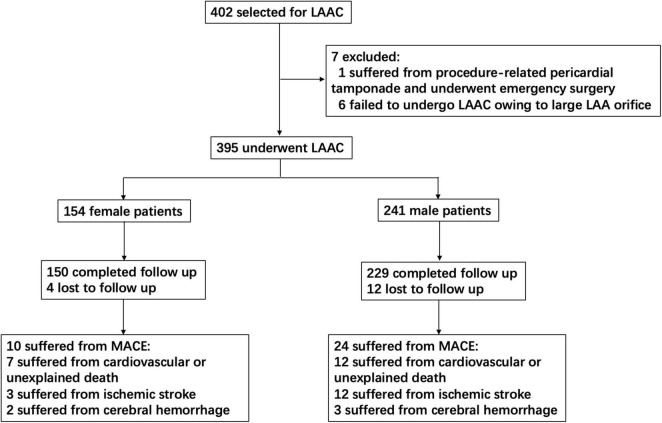
Study flow diagram. This chart showed patients flow regarding enrollment, follow-up, and MACE assessed for the study. LAAC, left atrial appendage closure; MACE, major adverse cardiovascular events.

From 2014 to 2018, 395 consecutive patients underwent LAAC in our center, including 154 women and 241 men (Figure 1). A total of 395 LAAC devices were implanted, including 339 Watchman, 52 ACP, 3 LAmbre, and 1 Lefort.

Compared with men, women were older (68.1 ± 7.9 vs. 64.6 ± 8.8, *p* < 0.01**), with higher average CHA2DS2-VASc score (4.0 ± 1.7 vs. 3.0 ± 1.6, *p* < 0.01**). Age ≥ 65 years (66.9% vs. 55.2%, *p* < 0.05*) and CHA2DS2-VASc score ≥ 2 (94.8% vs. 78.0%, *p* < 0.01**) were also more common in women. However, CAD was more common in men (40.7% vs. 30.5%, *p* < 0.05*). Among women, 6 (3.9%) were paroxysmal AF, 101 (65.6%) had hypertension, 29 (18.8%) had diabetes mellitus, 43 (27.9%) had congestive heart failure, 2 (1.3%) had major bleeding history, 5 (3.2%) had abnormal renal function, and 65 (42.2%) had ischemic stroke/TIA history. These clinical characteristics were not significantly different between the two groups. Neither average HAS-BLED score nor BMI showed significant differences between women and men. In TTE measurements, women had smaller LVEdD (47.8 ± 4.6 mm vs. 50.5 ± 5.4 mm, *p* < 0.01**). However, neither LVEF nor LAD showed significant differences between the two groups. The LAA orifice and length measured by TEE were also comparable between women and men (Table 1).

**TABLE 1:** Baseline clinical characteristics.

	Female (*n* = 154)	Male (*n* = 241)	*P*	OR (95%CI)
Age (years)	68.1 ± 7.9	64.6 ± 8.8	0.000**	−
Age ≥ 65 years old n (%)	103 (66.9%)	133 (55.2%)	0.021*	1.640 (1.077, 2.498)
BMI (kg/m^2^)	27.4 ± 13.5	26.2 ± 3.5	0.462	−
Paroxysmal AF *n* (%)	6 (3.9%)	4 (1.7%)	0.293	2.402 (0.667, 8.654)
Hypertension *n* (%)	101 (65.6%)	154 (63.9%)	0.733	1.077 (0.705, 1.645)
Diabetes mellitus *n* (%)	29 (18.8%)	35 (14.5%)	0.257	1.365 (0.796, 2.343)
Ischemic stroke/TIA history *n* (%)	65 (42.2%)	108 (44.8%)	0.611	0.899 (0.598, 1.353)
Coronary artery disease *n* (%)	47 (30.5%)	98 (40.7%)	0.041*	0.641 (0.418, 0.984)
Congestive heart failure *n* (%)	43 (27.9%)	56 (23.2%)	0.295	1.280 (0.806, 2.031)
Abnormal renal function *n* (%)	5 (3.2%)	7 (2.9%)	1.000	1.122 (0.350,3.599)
Abnormal liver function *n* (%)	5 (3.2%)	3 (1.2%)	0.312	2.662 (0.627,11.303)
Major bleeding history *n* (%)	2 (1.3%)	8 (3.3%)	0.358	0.383 (0.080, 1.829)
CHA2DS2-VASc score	4.0 ± 1.7	3.0 ± 1.6	0.000**	−
CHA2DS2-VASc score ≥ 2 *n* (%)	146 (94.8%)	188 (78.0%)	0.000**	5.145 (2.372,11.159)
HAS-BLED score	1.8 ± 1.1	1.9 ± 1.2	0.823	−
HAS-BLED score ≥ 3 *n* (%)	38 (24.7%)	72 (29.9%)	0.261	0.769 (0.486, 1.216)
LAD (mm)	49.0 ± 5.6	48.9 ± 5.5	0.859	−
LVEF (%)	52.8 ± 4.2	52.1 ± 5.8	0.744	−
LVEdD (mm)	47.8 ± 4.6	50.5 ± 5.4	0.000**	−
LAA orifice by TEE (mm)	20.9 ± 3.2	20.7 ± 3.3	0.674	−
LAA length by TEE (mm)	30.1 ± 6.0	30.8 ± 6.2	0.300	−

BMI, body mass index; AF, atrial fibrillation; TIA, transient ischemic attack; LAD, left atrial diameter; LVEF, left ventricular ejection fraction; LVEdD, left ventricular end diastolic diameter; LAA, left atrial appendage; TEE, transesophageal echocardiography; OR, odds ratio; CI, confidence interval; *P < 0.05; **P < 0.01.

### Procedure and In-Hospital Complications Characteristics

The LAA devices were successfully implanted in 151 (98.1%) women and 241 (100%) men. A total of three female patients had major residual leakages (> 5 mm), and the operations were considered unsuccessful. At postoperative 45-day follow-up, TEE showed that one patient’s major leakage decreased to less than 5 mm, whereas the other two major leakages remained unchanged. A number of two male patients suffered from procedure-related ischemic strokes, who both responded well to therapy. No female patient suffered from procedure-related ischemic stroke. A number of one female patient suffered from pericardial tamponade when the operator prepared to end the operation. Emergency pericardium puncture and medication rescued the patient. No male patient suffered from pericardial tamponade. Overall, there were not statistical differences in successful closure, procedure-related ischemic stroke, and pericardial tamponade between the two groups. There was no device displacement or procedure-related mortality in both groups. However, the device type, residual leakage, and short anticoagulants after LAAC were comparable between the two groups (Table 2).

**TABLE 2:** Procedure and in-hospital complications characteristics.

	Female (*n* = 154)	Male (*n* = 241)	*P*	OR (95%CI)
Successful closure *n* (%)	151 (98.1%)	241 (100%)	0.114	−
Device type *n* (%)			0.627	−
Watchman	133 (86.4%)	206 (85.5%)		
ACP	19 (12.3%)	33 (13.7%)		
LAmbre	1 (0.6%)	2 (0.8%)		
Lefort	1 (0.6%)	0		
Procedure-related ischemic stroke *n* (%)	0	2 (0.8%)	0.523	−
Pericardial tamponade *n* (%)	1 (0.6%)	0	0.390	−
Residual leakage *n* (%)	42 (27.5%)	66 (27.4%)	0.989	1.003 (0.637, 1.580)
Leakage size (mm)	0.7 ± 1.5	0.6 ± 1.0	0.854	−
Anticoagulants after LAAC *n* (%)			0.520	−
Warfarin	63 (40.9%)	107 (44.4%)		
NOACs	79 (51.3%)	112 (46.5%)		
DAPT	8 (5.2%)	10 (4.1%)		
other	4 (2.6%)	12 (5.0%)		

*ACP, Amplatzer Cardiac Plug; LAAC, left atrial appendage closure; NOACs, novel oral anticoagulants; DAPT, dual antiplatelet therapy; OR, odds ratio; CI, confidence interval.*

### Transesophageal Echocardiography Follow-Up at Postoperative 45 Days

A total of 331 patients (83.8%, including 132 women and 199 men) received TEE examination at postoperative 45 days. Female patients had higher peri-device leakage rate (57.6% vs. 39.2%, *p* < 0.01**), and most of the leakages were minor (< 5 mm). A number of two women had major leakages (> 5 mm) and continued their anticoagulation therapy. No male patient had major leakage. A number of three women had DRTs and responded well to anticoagulation therapy. A total of four men had DRTs. But two men did not respond well to the anticoagulation therapy, and DRTs still existed at 1-year outpatient visits. Then, one stopped anticoagulation therapy by himself at postoperative 1 year and 5 months. According to the 2-year phone follow-up, the patient did not suffer from thromboembolic event. The other patient was not willing to prescribe anticoagulation therapy at postoperative 1.5 years, and aspirin was replaced then. According to the 4-year phone follow-up, the patient did not suffer from thromboembolic event after postoperative 45 days (the patient suffered from ischemic stroke at postoperative 1 month and responded well to therapy). Neither major leakage nor DRT showed significant differences between the two groups (Table 3).

**TABLE 3:** Results of TEE follow-up at postoperative 45 days.

	Female (*n* = 132)	Male (*n* = 199)	*P*	OR (95%CI)
Residual leakage *n* (%)	76 (57.6%)	78 (39.2%)	0.001**	2.105 (1.346, 3.293)
Major leakage (> 5 mm) *n* (%)	2 (1.5%)	0	0.158	−
Minor leakage (≤ 5 mm) *n* (%)	74 (56.1%)	78 (39.2%)	0.003**	1.979 (1.267, 3.092)
Leakage size (mm)	1.4 ± 1.6	0.9 ± 1.3	0.001**	−
Device-related thrombosis *n* (%)	3 (2.3%)	4 (2.0%)	1.000	1.134 (0.250, 5.149)

TEE, transesophageal echocardiography; OR, odds ratio; CI, confidence interval; **P < 0.01.

### Complication Characteristics at Postoperative Mean 1,566 Days

A total of 379 patients (95.9%) completed the follow-up, including 150 women and 229 men. The average follow-up duration was 1,566 ± 534 days (ranging from 186 to 2,488 days). A total of 19 patients (7 women and 12 men) died from cardiovascular or unexplained death. There were 15 ischemic strokes (3 women and 12 men), 2 patients (1 woman and 1 man) died from ischemic stroke, and 7 patients (1 woman and 6 men) did not respond well to the therapy, leaving cognitive impairment or limb hemiplegia. A total of 5 patients (2 women and 3 men) had major bleeding events. Then, one female patient suffered from cerebral hemorrhage and died finally. A number of two male patients suffered from cerebral hemorrhage and did not respond well to the therapy, leaving visual barrier, language barrier, or limb hemiplegia. Altogether, 39 MACEs occurred in 34 patients (including 10 women and 24 men) (Figure 1).

**FIGURE 2:**
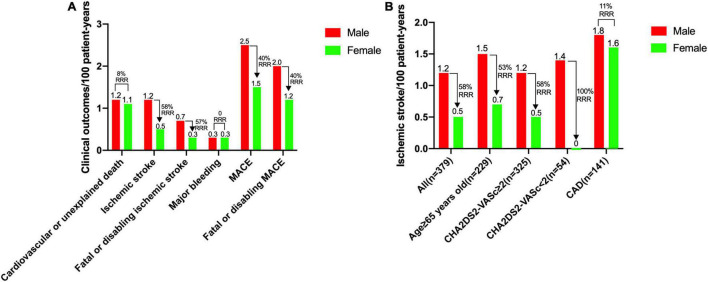
Rates of clinical outcomes between female and male patients. **(A)** Rates for cardiovascular or unexplained death, ischemic stroke, fatal or disabling ischemic stroke, major bleeding, MACE and fatal or disabling MACE. **(B)** Rates of ischemic stroke for overall population, patients with age ≥ 65 years, patients with CHA2DS2-VASc score ≥ 2, patients with CHA2DS2-VASc score < 2, and patients with a history of CAD. CAD, coronary artery disease; MACE, major adverse cardiovascular events, including cardiovascular or unexplained death, ischemic stroke or major bleeding; RRR, relative risk reduction.

The overall rate of ischemic stroke was 0.9 per 100 patient-years, and the overall rate of MACE was 2.1 per 100 patient-years. The ischemic stroke and fatal or disabling ischemic stroke rates in female patients were significantly lower than those in male patients (RRR 58 and 57%, respectively). Rates of cardiovascular or unexplained death and major bleeding were comparable between female and male patients (Figure 2A). On subgroup analyses of ischemic stroke, the reductions in patients with age ≥ 65 years (229 patients, men 1.5 vs. women 0.7 per 100 patient-years, RRR 53%) and patients with CHA2DS2-VASc score ≥ 2 (325 patients, men 1.2 vs. women 0.5 per 100 patient-years, RRR 58%) were larger. The rate of ischemic stroke in male patients with CHA2DS2-VASc score < 2 was 1.4 per 100 patient-years. However, no ischemic stroke was observed in any female patient with CHA2DS2-VASc score < 2. The RRR of ischemic stroke between female and male patients with CAD was only 11% (Figure 2B).

**FIGURE 3:**
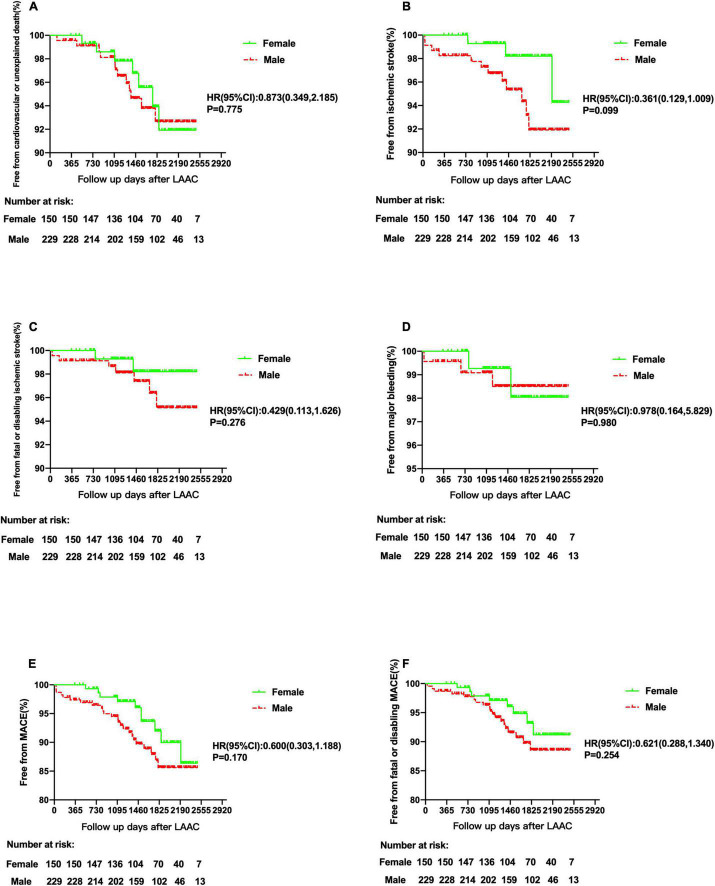
Rates of free from cardiovascular or unexplained death, ischemic stroke, fatal or disabling ischemic stroke, major bleeding, MACE, and fatal or disabling MACE between female and male patients. Kaplan–Meier curves of cardiovascular or unexplained death **(A)**, ischemic stroke **(B)**, fatal or disabling ischemic stroke **(C)**, major bleeding **(D)**, MACE **(E)**, and fatal or disabling MACE **(F)** according to female and male patients. MACE, major adverse cardiovascular events, including cardiovascular or unexplained death, ischemic stroke or major bleeding; LAAC, left atrial appendage closure; HR, hazard ratio; CI, confidence interval.

Kaplan–Meier analyses showed that cardiovascular or unexplained death and major bleeding were comparable between women and men. Compared with men, women had lower rates of ischemic stroke, fatal or disabling ischemic stroke, MACE, and fatal or disabling MACE, but none of them reached statistical differences (**HR:** 0.361, ***p*** = 0.099, **HR**:0.429, ***p*** = 0.276, **HR:** 0.600, ***p*** = 0.170, and **HR:** 0.621, ***p*** = 0.254, respectively) (Figure 3).

**FIGURE 4:**
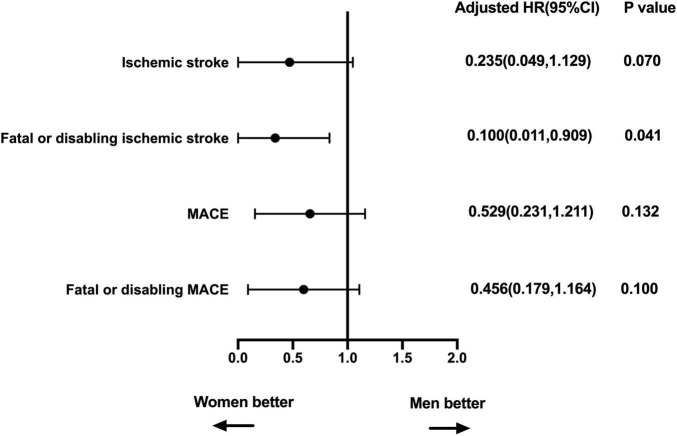
Multivariable-adjusted association of sex and clinical outcomes. Adjusted for CAD, CHA2DS2-VASc, LVEdD, and unsuccessful closure. MACE, major adverse cardiovascular events, including cardiovascular or unexplained death, ischemic stroke or major bleeding; HR, hazard ratio; CI, confidence interval; CAD, coronary artery disease; LVEdD, left ventricular end diastolic diameter.

Coronary artery disease, CHA2DS2-VASc, LVEdD, and unsuccessful closure were enrolled in the adjusted analyses for ischemic stroke, fatal or disabling ischemic stroke, MACE, and fatal or disabling MACE. The LVEdD for MACE and CAD for fatal or disabling MACE did not satisfy the proportional hazards assumption, and the other covariates all satisfied the proportional hazards assumption. In the multivariate Cox regression model, women had lower fatal or disabling ischemic stroke rate after LAAC implantation compared to men (**HR:** 0.100, ***p*** = 0.041) (Figure 4). In addition, on subgroup analysis of ischemic stroke in patients with age ≥ 65 years, multivariate Cox regression analysis showed that the clinical outcomes were comparable between women and men (adjusting for the confounding factors including CAD, CHA2DS2-VASc score, HAS-BLED score, BMI, and unsuccessful closure) (Table 4).

**TABLE 4:** Multivariate Cox regression analyses between sex and outcomes in patients with age ≥ 65 years old.

	Unadjusted		Adjusted*	
	HR (95%CI)	*P*	HR (95%CI)	*P*
Cardiovascular or unexplained death	1.177 (0.378, 3.663)	0.778	−	−
Ischemic stroke	0.427 (0.131, 1.395)	0.193	1.935 (0.326, 11.484)	0.468
Fatal or disabling ischemic stroke	0.483 (0.109, 2.138)	0.373	4.042 (0.380, 43.000)	0.247
Major bleeding	0.573 (0.059, 5.539)	0.645	0.289 (0.017, 4.998)	0.394
MACE	0.782 (0.339, 1.807)	0.569	−	−
Fatal or disabling MACE	0.747 (0.296, 1.888)	0.545	1.853 (0.581, 5.906)	0.297

MACE, major adverse cardiovascular events, including cardiovascular or unexplained death, ischemic stroke and major bleeding; *Adjusted for CAD, CHA2DS2-VASc score, HAS-BLED score, BMI and unsuccessful closure; BMI, Body Mass Index; CAD, Coronary Artery Disease; HR, Hazard Ratio; CI, confidence interval.

## Discussion

The main findings of this study are as follows: (1) LAAC can be completed with high success rate and low complications rates in both genders. (2) The ischemic stroke, cardiovascular or unexplained death, and major bleeding were comparable between women and men after LAAC implantation. (3) Compared with men, women had lower fatal or disabling ischemic stroke rate after LAAC implantation.

Female patients with NVAF have higher stroke risk, poorer outcome after stroke attack, and less likely to be treated with anticoagulation therapy. It has been well established that female patients with NVAF are older and more susceptible to stroke than male patients (4, 5). Female gender is related to higher mortality and poorer long-term outcome after stroke attack (1, 6). Functional outcomes at discharge and 6 months after stroke attack are significantly worse in female patients (7). Furthermore, female patients are significantly less likely to be treated with anticoagulation therapy at all levels of the CHA2DS2-VASc score (2).

Anticoagulation therapy is a way to prevent stroke in patients with AF, but women responding to anticoagulation therapy are different from men. A meta-analysis reported that female patients treated with warfarin had significantly higher residual stroke risk than male patients with NVAF, and the major bleeding risk on warfarin was comparable between women and men. Unlike warfarin, NOACs reduced stroke risk equally in male and female patients, and women on NOACs had less major bleeding risk than men (8). Another meta-analysis reported that female patients were benefitted more from prevention of major bleeding events, and male patients were benefitted more from prevention of stroke or systemic embolic events, when patients with AF were treated with NOACs (9). However, one meta-analysis reported no gender-related difference in the efficacy and safety of NOACs for patients with AF (10). Another meta-analysis also reported that major bleeding risk on anticoagulation therapy (including warfarin and NOACs) was not different between female and male patients (11).

Left atrial appendage closure has emerged as an alternative for OACs in stroke prevention among patients with NVAF, but the relationship between sex differences and the mid- and long-term efficacy and safety of LAAC has been still under investigation. Both the initiation and adherence to anticoagulation treatment among Chinese patients with NVAF have been much lower than that in US and European countries, especially among female patients with NVAF (12). LAAC has emerged as an alternative to anticoagulation therapy in the recent years, which showed similar effect in stroke prevention compared with warfarin or NOACs (13, 14). Although gender is an important factor in managing patients with NVAF, there are few studies that compare the efficacy and safety of LAAC between male and female patients. Then, one study reported that LAAC was an effective and safe strategy for patients with NVAF of both genders, and there was no sex difference in preventing thromboembolism events and decreasing bleeding risks after an average 2-year follow-up (15). The ischemic stroke, cardiovascular or unexplained death, and major bleeding were also comparable between women and men after LAAC implantation in our study. However, women had lower fatal or disabling ischemic stroke rate after LAAC implantation. It is interesting to postulate that women might benefit more than men do from LAAC. The small sample and low event rate may decrease the confidence of the conclusion, and further study is prompted to determine the gender-related differences in LAAC efficacy and safety.

Many devices related to LAAC have been appeared at present, but different results of different closure devices are lacking. The Amulet IDE trial, which was the only randomized controlled trial to compare Amulet and Watchman devices in stroke prevention, reported that the effectiveness and safety of Amulet device were non-inferior to the Watchman device in stroke prevention for patients with NVAF at 18-month follow-up. But procedure-related complications were higher in Amulet device than in Watchman device (16). Saad et al. also reported that periprocedural complications were higher in ACP device than in Watchman device. But the efficacy and safety between Watchman and ACP devices were comparable during the long-time follow-up (median follow-up durations of Watchman and ACP devices were 238 and 160 days, respectively) (17). The efficacy and safety were also comparable between Watchman and ACP devices during our long-term follow-up, but procedure-related complications were comparable between Watchman and ACP devices in our center (Supplementary Figure 1). However, the sample number of ACP device was small which may decrease the confidence of the conclusion, and further study is prompted to determine the differences in LAAC efficacy and safety in different devices.

Another interesting finding is that women were more prone to have peri-device leakage than men. During the implantation, the leakage rate was similar between men and women. While our TEE follow-up at postoperative 45 days revealed that women showed a much higher leakage rate than men did, the mechanism is not clear yet. Female patients with NVAF were generally older than male patients with NVAF. Both age and female gender have been related to more prominent atrial fibrosis among patients with atrial fibrillation (4). Atrial remodeling following AF reduced LAA contraction and elasticity, which might be responsible in reducing the stability of the LAAC device, thus increasing peri-device leakage among women. Anyway, the average leakage size remained below 5 mm, and the main residual leakage was minor leakage. Previous study has shown that minor peri-device leakage was not related to higher embolism risk after LAAC procedure (18), and our data corroborated with the previous study.

Ischemic stroke after LAAC is largely associated with DRT. There have been some studies indicating that DRT was related to ischemic stroke or thromboembolic complications (19, 20). Some studies reported that female gender was an independent predictor of DRT after LAAC (21, 22). However, one meta-analysis showed no difference in DRT between female and male patients (23). In our study, women had lower fatal or disabling ischemic stroke tendency, but DRT was comparable between the two groups. Some studies reported that DRT was not common at postoperative 45 days and increased at postoperative 6 and 12 months (24, 25). The DRT formation was mainly estimated with TEE at postoperative 45 days in our center, and the TEE data at postoperative 6 months and 1 year were incomplete (only a few patients accomplished TEE examination), which may underestimate the real incidence of DRT formation after LAAC implantation. Further study is needed to clarify the relationship between DRT and gender in LAAC implantation.

### Study Limitations

This is a retrospective and single-center study. The DRT formation was mainly estimated with TEE at postoperative 45 days in our center, and the TEE data at postoperative 6 months and 1 year were incomplete, which may underestimate the real incidence of DRT formation after LAAC implantation. Besides, some patients only completed 1, 2, 3, or 4- year follow-up, and the later following follow-ups were lost because they changed their telephone number. Finally, the small sample and low event rate may decrease the confidence of the conclusion. This finding remains to be tested with prospective, multicenter, and large sample trials.

## Conclusion

Left atrial appendage closure was feasible and safe for patients of both genders. The ischemic stroke, cardiovascular or unexplained death, and major bleeding were comparable between women and men. However, women were the independent protective factors against fatal or disabling ischemic stroke after LAAC implantation. Further studies are prompted to verify the findings and devise better strategies in the management of both genders.

## Data Availability Statement

The raw data supporting the conclusions of this article will be made available by the authors, without undue reservation.

## Ethics Statement

Ethical review and approval was not required for the study on human participants in accordance with the local legislation and institutional requirements. Written informed consent for participation was not required for this study in accordance with the national legislation and the institutional requirements.

## Author Contributions

YuC: study design, collecting data, statistical analysis, and writing of the manuscript. YaC and XS: study design and data interpretation. YZ, LQ, and CC: collecting data. All authors contributed to the article and have approved the final version.

## Conflict of Interest

The authors declare that the research was conducted in the absence of any commercial or financial relationships that could be construed as a potential conflict of interest.

## Publisher’s Note

All claims expressed in this article are solely those of the authors and do not necessarily represent those of their affiliated organizations, or those of the publisher, the editors and the reviewers. Any product that may be evaluated in this article, or claim that may be made by its manufacturer, is not guaranteed or endorsed by the publisher.

## Supplementary Material

Supplementary Figure 1 Rates of free from cardiovascular or unexplained death, ischemic stroke, fatal or disabling ischemic stroke, major bleeding, MACE, and fatal or disabling MACE between Watchman and ACP. Kaplan–Meier curves of cardiovascular or unexplained death **(A)**, ischemic stroke **(B)**, fatal or disabling ischemic stroke **(C)**, major bleeding **(D)**, MACE **(E)**, and fatal or disabling MACE **(F)** according to Watchman and ACP. MACE, major adverse cardiovascular events, including cardiovascular or unexplained death, ischemic stroke or major bleeding; LAAC, left atrial appendage closure; ACP, Amplatzer Cardiac Plug; HR, hazard ratio; CI, confidence interval.
