# Supplementary figures and images for: Sex Differences in Efficacy and Safety After Left Atrial Appendage Closure: A 4.3-Year Follow-Up Analysis
Source: Front Cardiovasc Med. 2022 May 18;9:814958. doi: 10.3389/fcvm.2022.814958 (PMC9157540; doi:10.3389/fcvm.2022.814958)

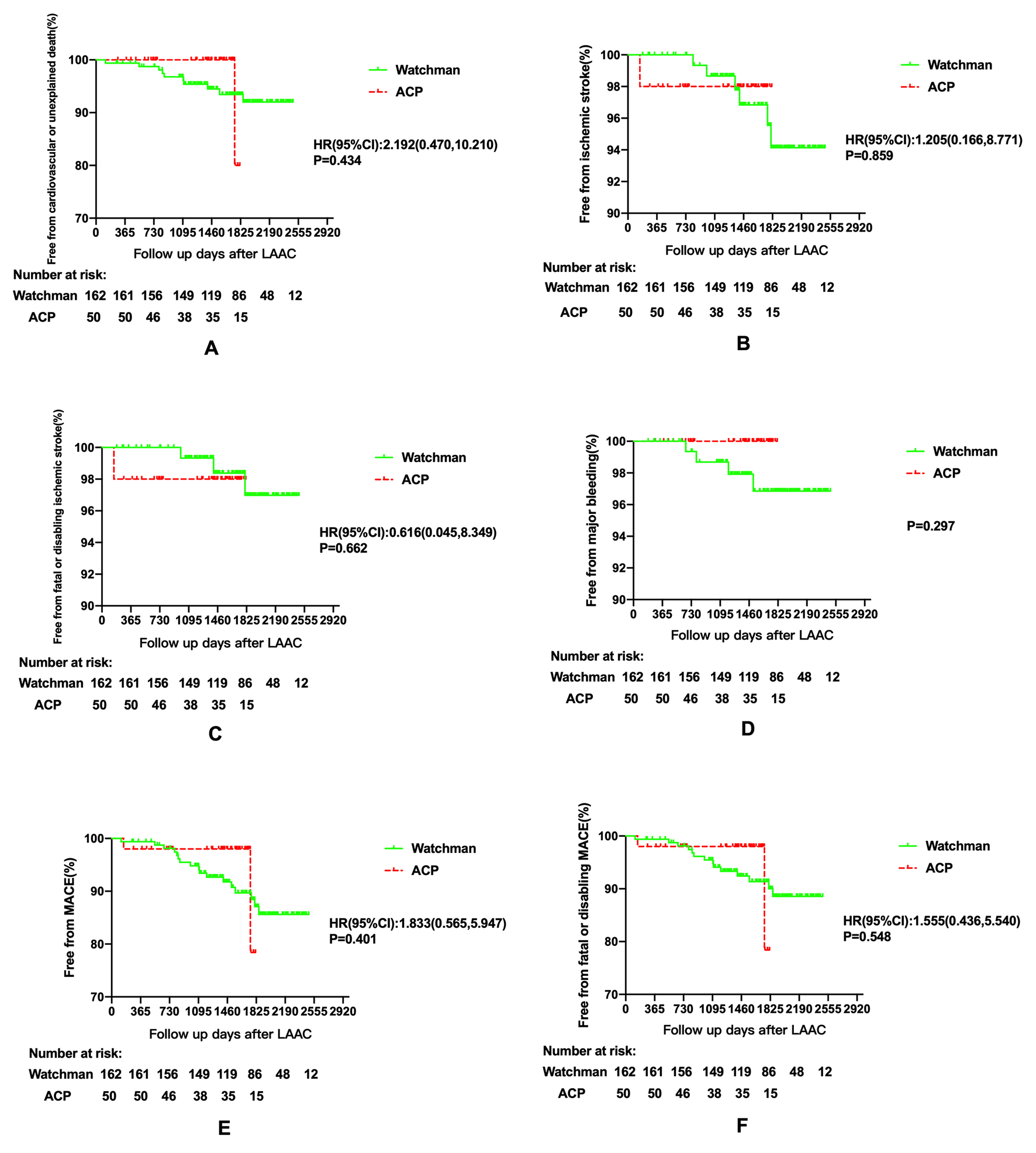

Supplement: Supplementary Figure 1 — Rates of free from cardiovascular or unexplained death, ischemic stroke, fatal or disabling ischemic stroke, major bleeding, MACE, and fatal or disabling MACE between Watchman and ACP. Kaplan–Meier curves of cardiovascular or unexplained death (A), ischemic stroke (B), fatal or disabling ischemic stroke (C), major bleeding (D), MACE (E), and fatal or disabling MACE (F) according to Watchman and ACP. MACE, major adverse cardiovascular events, including cardiovascular or unexplained death, ischemic stroke or major bleeding; LAAC, left atrial appendage closure; ACP, Amplatzer Cardiac Plug; HR, hazard ratio; CI, confidence interval. [file Image_1.TIFF]
