# Supplementary material for: Sex Differences in Efficacy and Safety After Left Atrial Appendage Closure: A 4.3-Year Follow-Up Analysis
Source: Front Cardiovasc Med. 2022 May 18;9:814958. doi: 10.3389/fcvm.2022.814958 (PMC9157540; doi:10.3389/fcvm.2022.814958)
Supplement: Supplementary file 2 [file Table_1.docx]

**Table S1. Multivariate Cox regression analyses between sex and outcomes**

|  | Unadjusted  HR (95%CI) | *P* | Adjusted^*^  HR (95%CI) | *P* |
| --- | --- | --- | --- | --- |
| Cardiovascular or unexplained death | 0.873(0.349,2.185) | 0.775 | - | - |
| Ischemic stroke | 0.361(0.129,1.009) | 0.099 | 0.235(0.049,1.129) | 0.070 |
| Fatal or disabling ischemic stroke | 0.429(0.113,1.626) | 0.276 | 0.100(0.011,0.909) | 0.041* |
| Major bleeding | 0.978(0.164,5.829) | 0.980 | - | - |
| MACE | 0.600(0.303,1.188) | 0.170 | 0.529(0.231,1.211) | 0.132 |
| Fatal or disabling MACE | 0.621(0.288,1.340) | 0.254 | 0.456(0.179,1.164) | 0.100 |

**Key:** MACE: major adverse cardiovascular events, including cardiovascular or unexplained death, ischemic stroke and major bleeding; *Adjusted for CAD, CHA2DS2-VASc, LVEdD and unsuccessful closure; LVEdD: left ventricular end diastolic diameter; CAD: Coronary artery disease; HR: Hazard Ratio; CI: confidence interval; *: *P*<0.05.
